# Capabilities and limitations of 3D printed microserpentines and integrated 3D electrodes for stretchable and conformable biosensor applications

**DOI:** 10.1038/s41378-019-0129-3

**Published:** 2020-04-20

**Authors:** Charles Didier, Avra Kundu, Swaminathan Rajaraman

**Affiliations:** 1 Nanoscience Technology Center (NSTC), University of Central Florida, Orlando, FL 32826 USA; 2 Burnett School of Biomedical Sciences, University of Central Florida, Orlando, FL 32827 USA; 3 Department of Materials Science & Engineering, University of Central Florida, Orlando, FL 32816 USA; 4 Department of Electrical & Computer Engineering, University of Central Florida, Orlando, FL 32816 USA

**Keywords:** Electrical and electronic engineering, Electronic properties and materials

## Abstract

We explore the capabilities and limitations of 3D printed microserpentines (µserpentines) and utilize these structures to develop dynamic 3D microelectrodes for potential applications in in vitro, wearable, and implantable microelectrode arrays (MEAs). The device incorporates optimized 3D printed µserpentine designs with out-of-plane microelectrode structures, integrated on to a flexible Kapton® package with micromolded PDMS insulation. The flexibility of the optimized, printed µserpentine design was calculated through effective stiffness and effective strain equations, so as to allow for analysis of various designs for enhanced flexibility. The optimized, down selected µserpentine design was further sputter coated with 7–70 nm-thick gold and the performance of these coatings was studied for maintenance of conductivity during uniaxial strain application. Bending/conforming analysis of the final devices (3D MEAs with a Kapton® package and PDMS insulation) were performed to qualitatively assess the robustness of the finished device toward dynamic MEA applications. 3D microelectrode impedance measurements varied from 4.2 to 5.2 kΩ during the bending process demonstrating a small change and an example application with artificial agarose skin composite model to assess feasibility for basic transdermal electrical recording was further demonstrated.

## Wearables: Stretchable microstructures for microelectrode sensors

A unique method to make wearable electronics using flexible microstructures have been explored and characterized by researchers in the United States. Wearable devices such as sensors or power generators require stable, stretchable electronics, otherwise their electrical performance will degrade when they are deformed. Swaminathan Rajaraman’s team at the University of Central Florida investigated the capabilities and performance of 3D-printed microserpentines; shapes engineered to be flexible. They used an analytical model to optimize the design of the microserpentine structures to be maximally flexible. Next, they applied varying thicknesses of nano-gold to the microserpentines to determine which thickness remained most stable when stretched. Finally, the team incorporated the microserpentines into a sensor device to take measurements from artificial skin. These findings demonstrate the potential of 3D-printed microserpentine structures for use in sensors and other wearable electronics.

## Graphical abstract

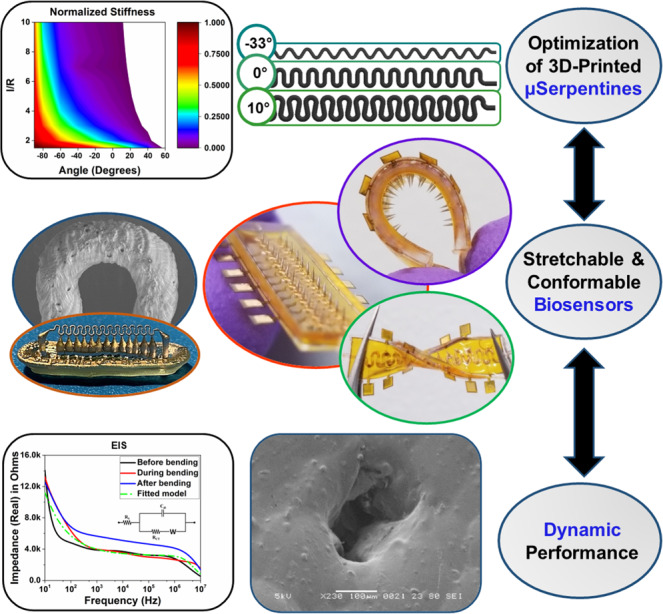

## Introduction

Stretchable electronics and microsensors have begun to be applied to several consumer and biomedical areas, including wearables for personal health monitoring^1,2^, surgical robotics^3^, implantable devices^4^, tactile sensors^5^, and devices for power harvesting and storage^6^. A basic requirement in the micro-structuring of such devices is the design and development of the components of the system that are able to mechanically deform without losing their ability to electrically function successfully. Inorganic materials used in the microfabrication of stretchable microsensors such as silicon^7^ and aluminum^8^ are very stiff and deform to an extent where electrical failure occurs at small amounts of tensile strain^9^. In order to alleviate this problem, a common strategy for a device design with such materials, is to replace “straight wire” features^10^ fabricated out of these materials with shapes engineered to be stretchable and flexible including “Archimedean spiral”^11^, “µserpentines,” and other geometries^6,12^. Specifically in flexible electronics devices, “serpentine” designs have resulted in enhanced strain performance^13^. In addition to the aforementioned standard materials, there are numerous material sets and combinations currently in use for the fabrication of stretchable electronics, with polydimethylsiloxane (PDMS) being a widely used substrate and packaging material^14,15^.

A common structure in a stretchable electronics system is microelectrode, which consists of a substrate (with an additional package or the package defined on the substrate) atop which a grid or line of metal traces and an insulation layer are defined^16^. Such two-dimensional (2D) and three-dimensional (3D) microelectrode arrays (MEAs) have become ubiquitous in in vitro, cell-based biosensing^17^, wearable^18^, implantable^19^, and environmental sensing applications^20^.

Recently, the ease of microfabrication of complex shapes such as µserpentines and base structures for 2D and 3D MEAs has been achieved through rapid and cost-effective additive manufacturing methods like 3D printing^1^. Owing to the commercial availability of various 3D printing systems and the innovations of makerspace environments, the development of 3D printed devices has increasingly expanded and continues to show promise in innovation^21^. While prior work demonstrating the development of 2D and 3D MEAs in static cell culture settings has been reported^22^ (including from our group), to date understanding the capabilities and limitations of 3D printed geometries and their application to *stretchable and dynamic* 3D microelectrodes is missing.

In this work, considerations and limitations for using standard and commercially available clear resin^23^ to produce a stretchable and flexible engineered design that can incorporate robust 3D structures through additive micro-stereolithographic (µSLA) 3D printing is explored. This work has adapted and expanded on the mathematical background on printed µserpentine structures that was recently developed^24^. Similarly, the metallization of such 3D printed structures has not been fully characterized or understood. To this end, metallized 3D printed µserpentines were analyzed for performance, reliability, and bending/conformance.

Beyond the optimization of metallized µserpentines, 3D MEA devices still require a package and an insulation. Materials such as polyimide (PI) and PDMS provide choices in polymeric backbone layers with improved mechanical match for dynamic biological tissue experiments (Young’s modulus of PI: 2.5 GPa^25^ and PDMS: 360 kPa–2.97 MPa^26,27^). Further, PDMS is commonly used in 2–2.5D flexible devices as both the substrate and the insulation material, because it also provides tunable mechanical and dielectric properties^28^. In this work, PDMS has been used as an elastomeric insulation and thin Kapton^®^ PI has been used as a packaging substrate.

**Fig. 1:**
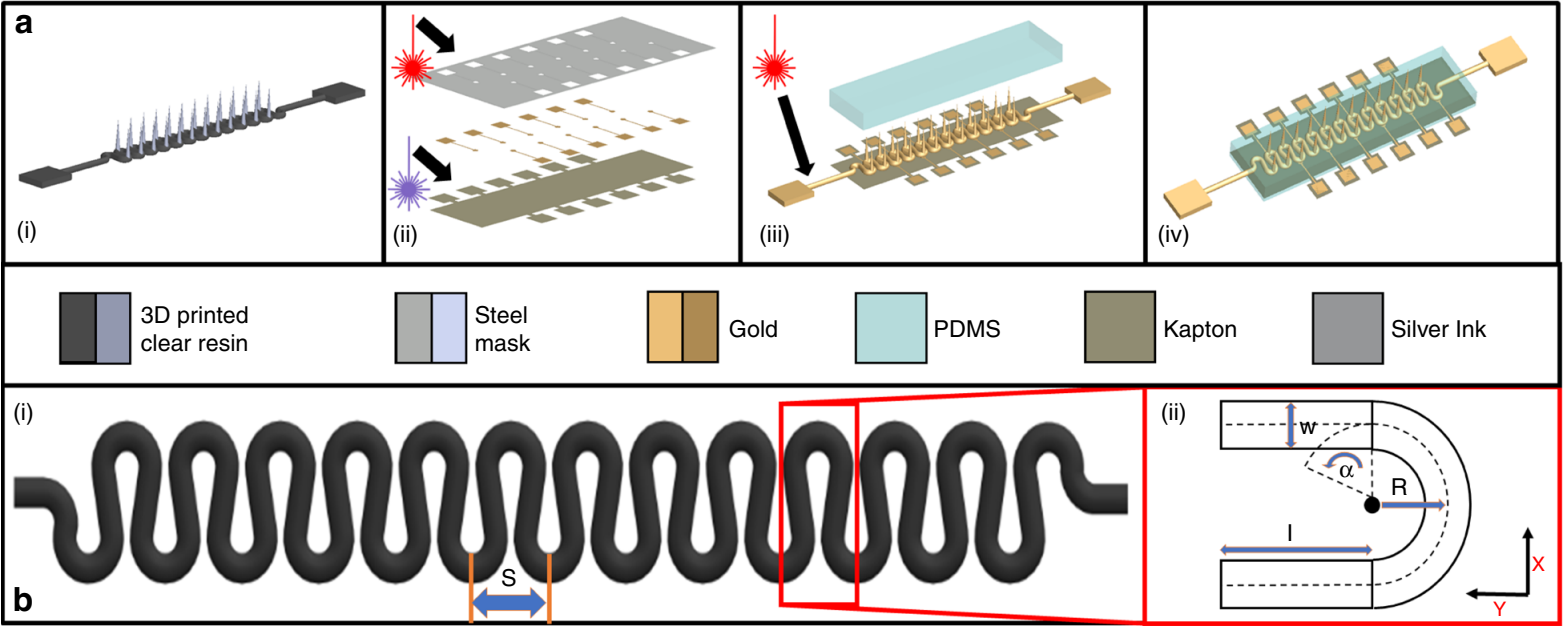
Schematic representation of the process flow for the dynamic 3D MEA fabrication with µserpentines and details of the µserpentines. **a** Schematic of the process flow. (i) 3D printed µserpentine with out-of-plane electrode structures. (ii) Initial fabrication steps, including the UV laser micromachining of the Kapton® substrate and the IR laser micromachining of the steel deposition mask with associated sputter metallization of the Au traces. (iii) Assembly of the full device, where a metallized µserpentine is IR laser micromachined selectively to isolate the electrodes and then is placed on the Kapton package and insulated with PDMS. (iv) Schematic of the fully assembled device. **b** Schematic of a µserpentine for illustration of the geometric parameters. (i) µserpentine denoting a singular “S” subunit and highlighting (ii) illustrates the various geometric parameters and reference orientation.

A schematic for the microfabrication and packaging of the 3D MEAs is depicted in Fig. 1a. The stretchable, conformable 3D MEA was constructed with a singular µserpentine design and was subsequently electrically and mechanically characterized. An analytical model was developed for the design of the printed µserpentine structures. Further, to demonstrate the potential applicability of this device in biopotential measurements, puncture and conductivity characteristics on an artificial skin agarose model were explored. Such a technology platform introduces a novel, flexible, rapidly fabricated, and cost-effective packaging substrate that can be applied to a variety of flexible biosensor, wearable, implantable, and cell/tissue culturing applications^2,29^.

## Results and discussion

Widlund et al. compiled the two key equations, which laid the foundation for analytically modeling µserpentine geometries and down selecting 3D printed test structures^24^. Their work combined and condensed plane-strain elastic theory^30^, as well as Winkler curved beam theory^31^ to produce Eqs. 1 and 2 as outlined below.

Equation 1 is the compound equation for the effective stiffness of a given µserpentine design:

1 $$\frac{{{\rm{PS}}}}{{2{\bar{\mathrm E}}wu_0}} = \frac{{\left( {\frac{w}{R}} \right) \ast \left( {\left( {{\mathrm{Cos}}\left[ \alpha \right]} \right) - \left( {\left( {\frac{l}{{\left( {2R} \right)}}} \right){\mathrm{Sin}}\left[ \alpha \right]} \right)} \right)}}{{\begin{array}{*{20}{c}} {2\left[ {\left( {\left( {{\mathrm{Cos}}\left[ \alpha \right]^2} \right)\left( {\left( {\frac{{l^3}}{{(2R^3)}}} \right) + \left( {\left( {3\left( {\frac{\pi }{2} + \alpha } \right)} \right)\left( {\frac{{l^2}}{{R^2}}} \right)} \right) + \left( {12\left( {\frac{l}{R}} \right)} \right) - 12\left( {\frac{\pi }{2} + \alpha } \right)} \right)} \right)} \right.}\\ { + \left( {{\mathrm{Sin}}\left[ {2\alpha } \right]\left( {\left( {6\left( {\frac{\pi }{2} + \alpha } \right)\left( {\frac{l}{R}} \right) + 9} \right)} \right)} \right)}\\ {\left. { + \left( {\left( {\left( {\left( {\frac{{w^2}}{R}} \right)\left( {\frac{\pi }{2} + \alpha } \right)\left( {\frac{l}{{2R}}{\mathrm{Cos}}\left[ \alpha \right] + {\mathrm{Sin}}\left[ \alpha \right]} \right)^2 + \left( {\left( {\frac{l}{{2R}}} \right)\left( {{\mathrm{Sin}}\left[ \alpha \right] + \left( {\left( {\frac{{3{\bar{\mathrm E}}}}{{2G}}} \right){\mathrm{Cos}}\left[ \alpha \right]} \right)} \right)} \right)} \right) + \left( {18\left( {\frac{\pi }{2}} \right) + \alpha } \right)} \right)} \right)} \right]}\end{array}}}$$

In this equation, “*P*” denotes the reaction force, “*S*” is the length of a given serpentine, “*w*” is the width of that serpentine, and “2*u*_0_” is the effective displacement of the serpentine, giving rise to ${\mathrm{PS}}/\left( {2{\bar{\mathrm E}}wu_{0}} \right)$ on the left side of the equation, which is expanded on the right side as determined in the work by Widlund et al.^24^.

**Fig. 2:**
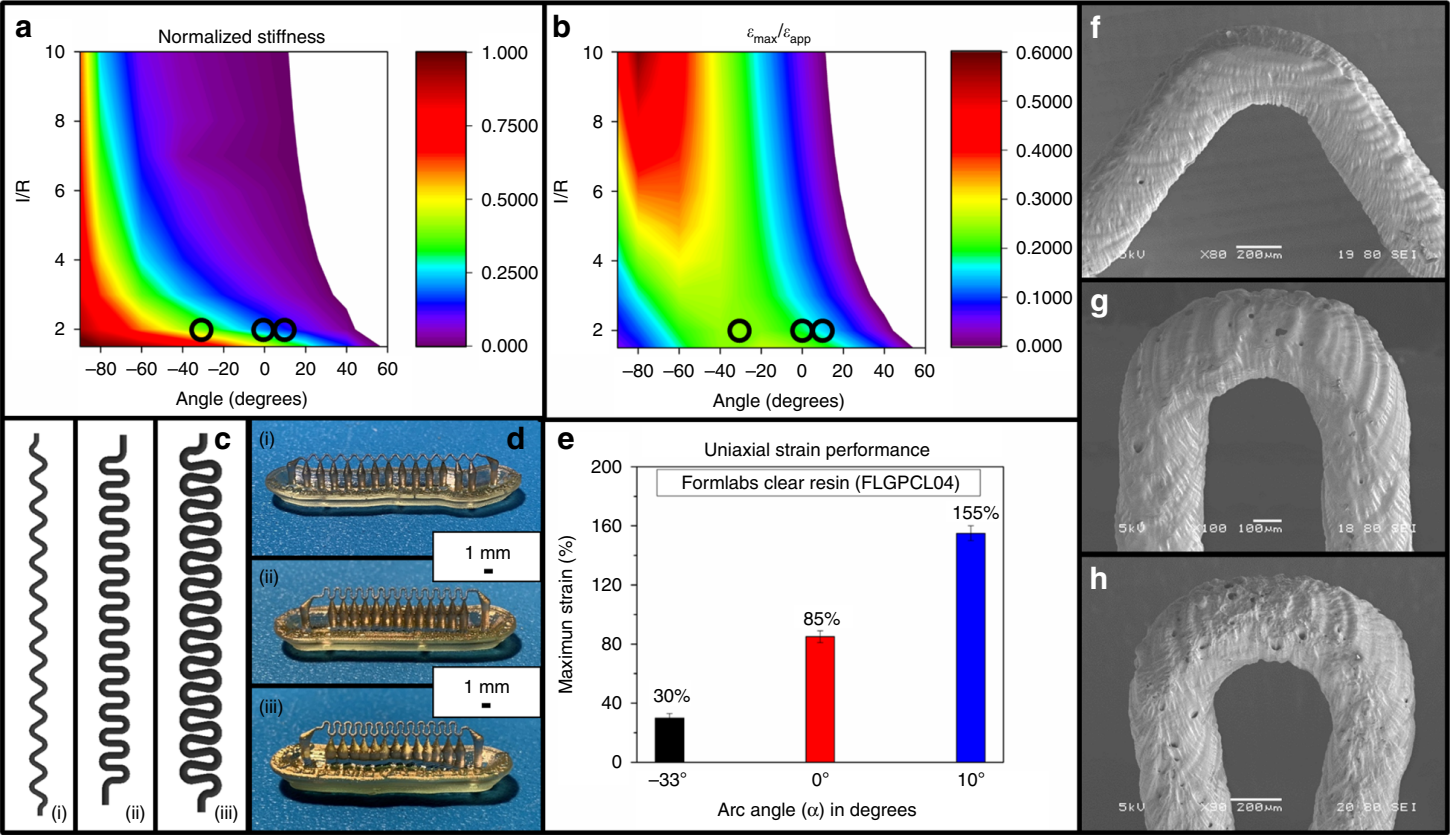
Graphical representations of the µserpentine modeling equations and simple strain data, with images of the printed µserpentines. **a** Contour plot of the normalized stiffness for a µserpentine (with Formlabs Clear resin) calculated from the analytical model developed using Eq. 1. The hotter colors denote conformation closer to the stiffness of a flat ribbon (where *α* = −90°). Circles indicate the design choices for this work. **b** Contour plot of the maximum effective strain on the inner U-bend of a µserpentine, calculated from the analytical model developed using Eq. 2. The lower values in this contour plot indicate a higher maximum effective strain that can be applied before failure. Circles indicate design choices for this work. **c** (i–iii) Schematic representations of the three chosen µserpentine designs, from *α* = −33° (i), to *α* = 0° (ii), and *α* = 10° (iii). **d** Optical micrographs of the µSLA 3D printed µserpentines after metallization corresponding to their schematics in **c**. **e** Experimentally measured maximum uniaxial strain of the three µserpentine designs, leading to the down selection of the *α* = 10° design. **f**–**h** SEM images of the three µserpentine designs corresponding to (i–iii) in **c**. The minor print defects seen from the µSLA printing process do not impact the design’s performance and are consistent across all prints.

Equation 2 is similarly the compound equation for the maximum effective strain on the inner U-bend curvature (Fig. 2b) of a given µserpentine design and is expanded from *ε*_max_ (the maximum tensile strain on the serpentine) and *ε*_applied_ (the effective applied tensile strain):

2 $$\frac{{\varepsilon [{\mathrm{max}}]}}{{\varepsilon [{\mathrm{app}}]}} = \frac{{\left( {\left( {\frac{w}{R}} \right)\left( {\left( {\frac{{12}}{{\left( {2 - \left( {\frac{w}{R}} \right)} \right)}}} \right) + \left( {\left( {\left( {\frac{{12}}{{\left( {2 - \left( {\frac{w}{R}} \right)} \right)}}} \right) - \left( {\frac{w}{R}} \right)} \right)\left( {{\mathrm{Sin}}\left[ \alpha \right] + \left( {\left( {\frac{l}{{2R}}} \right){\mathrm{Cos}}\left[ \alpha \right]} \right)} \right)} \right)} \right) \ast \left( {\left( {{\mathrm{Cos}}\left[ \alpha \right] - \left( {\left( {\frac{l}{{2R}}} \right){\mathrm{Sin}}\left[ \alpha \right]} \right)} \right)} \right)} \right)}}{{\begin{array}{*{20}{c}} {\left( {\left( {{\mathrm{Cos}}\left[ \alpha \right]^2} \right)\left( {\left( {\frac{{l^3}}{{\left( {2R^3} \right)}}} \right) + \left( {\left( {3\left( {\frac{\pi }{2} + \alpha } \right)} \right)\left( {\frac{{l^2}}{{R^2}}} \right)} \right) + \left( {12\left( {\frac{l}{R}} \right)} \right) - 12\left( {\frac{\pi }{2} + \alpha } \right)} \right)} \right)} \\ { + \left( {{\mathrm{Sin}}[2\alpha ]\left( {\left( {6\left( {\frac{\pi }{2} + \alpha } \right)\left( {\frac{l}{R}} \right) + 9} \right)} \right)} \right) + \left( {\left( {\left( {\left( {\frac{{w^2}}{R}} \right)\left( {\frac{\pi }{2} + \alpha } \right)\left( {\frac{l}{{\left( {2R} \right)}}{\mathrm{Cos}}\left[ \alpha \right] + {\mathrm{Sin}}\left[ \alpha \right]} \right)^2} \right.} \right.} \right.} \\ { + \left. {\left( {\left( {\frac{l}{{2R}}} \right)\left( {{\mathrm{Sin}}[\alpha ] + \left( {\left( {\frac{{3{\bar{\mathrm E}}}}{{2G}}} \right)\left. {{\mathrm{Cos}}[\alpha ]} \right)} \right)} \right)} \right) + \left( {18\left( {\frac{\pi }{2}} \right) + \left. \alpha \right)} \right)} \right)} \end{array}}}$$

Both equations were tabulated with respect to the specific aspects of a µserpentine interconnect geometry, as shown in Fig. 1b(ii). In these equations, close attention needs to be devoted to the *α* value and the ratio of *l*/*R*.

The resin used for the device in this work was the Clear (FLGPCL04) resin from Formlabs^23^. This material is inherently inflexible but could resolve the necessary structures to create 3D electrodes to be used in the final device and as a result was chosen as the material for 3D printing. The plane strain modulus of the resin material is denoted as “Ē,” and the shear modulus of the resin material is denoted as “*G*.” These values were calculated using the Young’s modulus of the Formlabs Form 2 Clear resin at 2.8 GPa^23^ and Poisson’s ratio of 0.4 (poly(methyl methacrylate))^32^, which is the closest approximation available for the resin, since it is largely proprietary but is known to be methacrylate based^23^.

The *α* values denote the degree of completeness of the central arc of the semi-circle of the µserpentine U-bend, with respect to a standard semi-circle (has an *α* value of 0°). All values of *α* are denoted in one quadrant of the central circular arc length and are reflected bilaterally across the semi-circle. The positive values of *α* denote a circle closer to completion. An *α* value of 0° being a standard semi-circle and an *α* of 90° being a completed circle. Values of *α* <0° denote a less than complete circle, and thus the structure would reach a flat and straight ribbon at an *α* of −90°. The length “*l*” is the distance between the U-bends of the µserpentine interconnects, and the ratio of this length to the radius “*R*” of the U-bend’s semi-circle, is an important distinction for the distribution of strains as the µserpentine is stretched. The width “*w*” was fixed for this analytical calculation at an experimentally defined value of 400 µm. This was the smallest dimension that the Form 2 µSLA 3D printer could resolve in this configuration because of the need for printing support structures. Similarly, the value of “*R*” was fixed at 400 µm to ensure maximum printability given the previous width constraint. It should be noted, that because of the µserpentine’s circular profile in this experimental set-up, the thickness and the width were set as equivalent, which differs from the original mathematical set-up of Widlund et al.^24^, in which the µserpentines were printed to have a rectangular cross-sectional profile, leading to differing width and thickness. The singular subunit for a given µserpentine design is also illustrated in Fig. 1b(i), as denoted by “*S*.”

Figure 2a, b, shows a contour plot of Eqs. 1 and 2. These contour plots represent the variation of normalized stiffness and *ε*_max_/*ε*_applied_, where the angle *α* was varied along the *x*-axis, and the ratio of *l*/*R* varied on the *y*-axis. In both the graphs, the quantity *w*/*R* was fixed for calculations because the width of the µserpentine never changed owing to the experimental constraint previously mentioned. Figure 2a was normalized with respect to the calculated values of the expected stiffness (Eq. 1) for an *α* of −90°, where the µserpentine would devolve into a flat ribbon and thus would be the stiffest conformation. Figure 2b is a similarly derived contour plot representing analytical calculations from Eq. 2, which denotes the maximum effective strain that would be applied to the inner curvature of the U-bends. The values denoted in white in this figure violate the “non-overlapping constraint” as outlined in Widlund et al.^24^, which represent designs that are mathematically and geometrically impossible. Printing at the smallest resolvable design conformation on a µSLA 3D printer has associated design challenges, which limit combinations of *l*/*R* and *α* that were chosen in this work. The region in Fig. 2a corresponding to a normalized stiffness between 0 and 0.25 represents the design conformations of a possible µserpentine, which would be the least stiff. While this might appear advantageous, theoretical and µSLA print constraints (including more scaffolding supports for longer “*l*” values) necessitate design choices that balance stiffness and effective strain.

Three designs were chosen and are denoted on the graph with black circles in Fig. 2a, b. All three of the variants had *l*/*R* ratios of 2 (with *l* = 800 µm) in order to ensure non-overlapping print configurations, as higher values of “*l*” would have resulted in fused µserpentines at *α* = 10°. As previously mentioned, there is a mutually exclusive relationship between the supporting scaffolds necessary to resolve µserpentines and µserpentines with a lower stiffness value. A minimum of one linear support per “*S*” unit along the *x*-axis (denoted in Fig. 1b(ii)) is necessary to resolve the *l*/*R* = 2 designs. Increasing the “*l*” value would increase scaffolding necessary for a “*S*” unit along the *y*-axis, to values >1. This would result in either fused prints or unstable printed structures, negating benefits that are theoretically possible. This limitation narrowed down the real variation in design stretchability and flexibility to the value of *α*, which in fact does contribute greatly to the overall performance of a particular design. Values for *α* at 0° and 10° were chosen to illustrate (according to the calculated theoretical data) that the small increase of 10° would significantly have a positive impact on the design (supported in Fig. 2b). The *α* = −33° was arbitrarily chosen in the negative region to be closer to the mid-range of stiffness values and to study negative design values of *α*. These decisions are supported by the analytical model for maximum strain which shows that these values for the inner U-bends are inversely related to the overall maximum stretchability of the µserpentine design^24^. Figure 2c(i–iii) demonstrate the 3D CAD renderings of the three chosen µserpentine designs, and Fig. 2d(i–iii) illustrate the optical micrographs of the 3D printed and metallized designs before release from their printing support structures. Figure 2e plots the effective maximum strain attained by the three designs experimentally (average *N* = 6). The *α* = −33° design had the poorest performance with failure at 30% increase in length during uniaxial strain testing. The *α* = 0° design performed largely better as expected, with the ability to extend up to a maximum of 85% additional strain from rest prior to failure.

The optimized design (*α* = 10°) as suggested by the analytical model and expected to outperform the other two designs was able to resist failure until a uniaxial strain of 155% was applied to the structure. This structure was chosen for further metallization optimization and device fabrication. Figure 2f, g shows scanning electron microscopic (SEM) images of the three designs, after printing. The striations and small defects in the resin surface are standard features of µSLA printing, by virtue of the laser-spot definition of individual printed layers, and did not have any significant impact on the performance of the µserpentine structures.

**Fig. 3:**
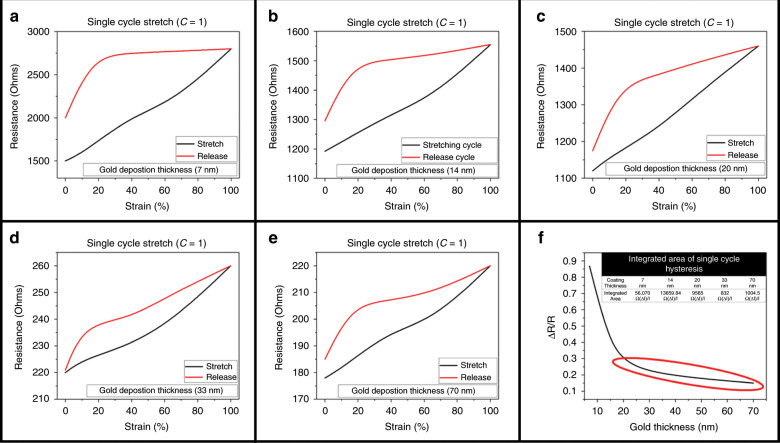
Single cycle hysteresis (strain and release) graphs for each of the Au coating thicknesses. **a** 7 nm coating hysteresis cycle. **b** 14 nm coating hysteresis cycle. **c** 20 nm coating hysteresis cycle. **d** 33 nm coating hysteresis cycle. **e** 70 nm coating hysteresis cycle. **f** Δ*R*/*R* for each of the coating thicknesses. The red highlighted area indicated coatings that are more suitable for consistent conduction performance over strain, including the 20, 33, and 70 nm coatings. The inset table lists the tabulated hysteresis integration areas, indicating the most (33 nm) and least (7 nm) consistent conduction performance over the single cycle.

To accomplish conductive µserpentines toward the goal of 3D stretchable microelectrodes, a conformal metal deposition technique was needed that could coat the striated surface of the µserpentine and maintain its integrity under strain. Sputter metal coating provides an ideal, easily accessible method to accomplish conformal metal coatings with precisely defined thicknesses^33^. Five different coating thicknesses (7, 14, 20, 33, and 70 nm) were assessed to obtain an optimal coating of sputtered gold, with resistance performance under strain as the measurand. Figure 3a–e shows the single cycle hysteresis for each of the five coating thicknesses, where the maximum strain value for the optimized *α* = 10° design never exceeded 100% uniaxial strain (well below the maximum value of 155%, Fig. 2e). The change in resistance to resistance at rest (Δ*R*/*R*) was calculated to see the reliability of each of the coatings during a single stretch cycle (Fig. 3a–e). Figure 3f shows a plot of the Δ*R*/*R* values from the previous graphs (Fig. 3a–e), and highlights that asymptotic region encompassing the 20, 33, and 70 nm coatings. This low variance in the resistance values indicates that this asymptotic range is the most suitable for consistent conduction values while the device is under strain. This is evident when examining the requirements for viable µserpentine strain sensors, which require a much higher Δ*R*/*R*^34^. The inset to Fig. 3f corroborates this finding, as the integrated *Ω*(Δ*l*)/*l* under the hysteresis curve are lowest for these three coatings as well: 7 nm corresponding to an integrated area of 56,070 *Ω*(Δ*l*)/*l*, 14 nm with an area of 13,659.84 *Ω*(Δ*l*)/*l*, 20 nm with an area of 9,565 *Ω*(Δ*l*)/*l*, 33 nm with an area of 832 *Ω*(Δ*l*)/l, and 70 nm with an area of 1,004.5 *Ω*(Δ*l*)/*l*. This indicates a much tighter strain to relaxation behavior for the thicker coatings, which would improve device stability and reliability over time. The 33 nm has the lowest area under such a hysteresis curve and was chosen as the most reliable value for device construction.

**Fig. 4:**
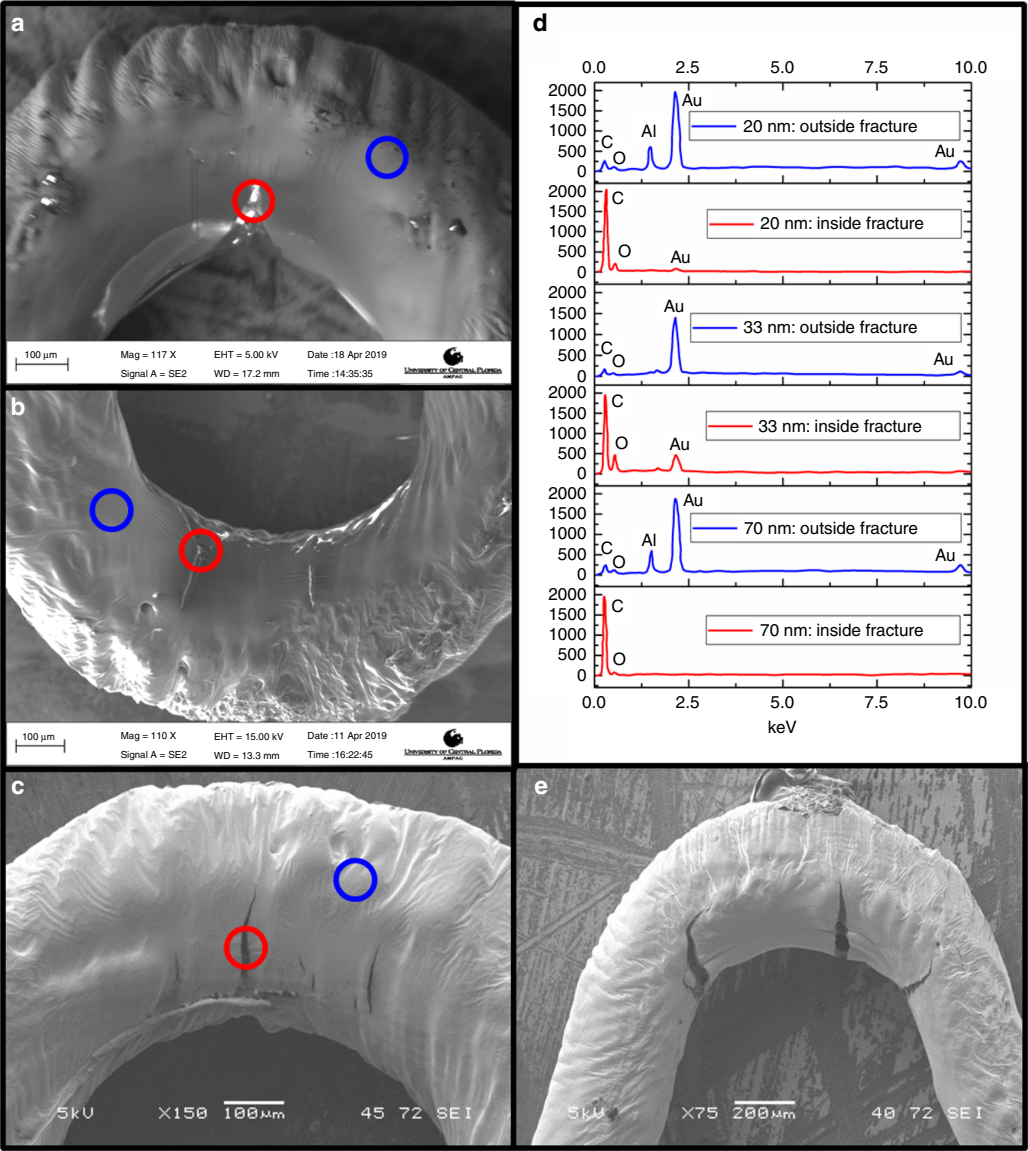
SEM and associated EDS data for analysis of optimized Au coating thickness after the application of uniaxial strain. In all the SEM images, the circles indicate points where EDS analysis was performed, with red being inside fracture points and blue being outside fracture points. **a** SEM of the 20 nm coating for EDS analysis. **b** SEM of the 33 nm coating for EDS analysis. **c** SEM of the 70 nm coating for EDS analysis. **d** Color corresponding EDS data for red and blue circles from each of the previous SEM images. Generally, more Au was located outside of the fracture as was expected, and the real difference in the analysis was the amount of Au that remained in the fracture. **e** SEM image of a separate 70 nm-thick Au coating on a µserpentine after the application of strain, demonstrating much large fracturing of the Au film.

To analyze fracture compositional changes in the coatings after the application of strain, energy dispersive X-ray spectroscopy (EDS) was performed on the coatings with the lowest area under such a hysteresis curve. Figure 4 depicts SEM images of the 20 nm (Fig. 4a), 33 nm (Fig. 4b), and 70 nm (Fig. 4c) U-bends and the associated fractures in the gold film from applied strain, after being subjected to one-cycle strain and relaxation. Each SEM has two highlighted sections for compositional comparison using EDS: the red circle denotes regions inside the fracture, and blue circle denotes regions in the unaffected gold film outside the fracture. The focus of the fracture study was placed on the inner curvature of the U-bend as informed by the analytical model since these areas would be areas of the highest strain concentration. This observation was conformed during experimental analysis of the structures after strain application under the SEM. The SEM images show fracture occurring in all three coating thicknesses under strain. However, upon releasing the strain it was expected that the coatings would behave differently, and it was hypothesized that the fracture composition of the 33 nm coating would provide clues to its behavior. Figure 4d shows the EDS data from the performed experiment. It is observed that, among all three coating thicknesses, gold is abundantly present outside the fracture point under strain. The significant differences occur when the EDS is performed inside the micro-fracture areas. While peaks for oxygen and carbon (denoting the resin underneath the gold) are observed in all three coatings, only the 33 nm thickness shows a significant amount of gold inside the micro-fracture region, relative to the other two coatings. This could explain the lower area under such a hysteresis curve for this coating. The aluminum peak in the EDS data is attributed to the background metal spectrum of the sample stage. Figure 4e is an SEM image, illustrating the sharp micro-fracture occurring in the 70 nm coating, which could explain the increased resistance observed once at the end of the strain application cycle in Fig. 3.

**Fig. 5:**
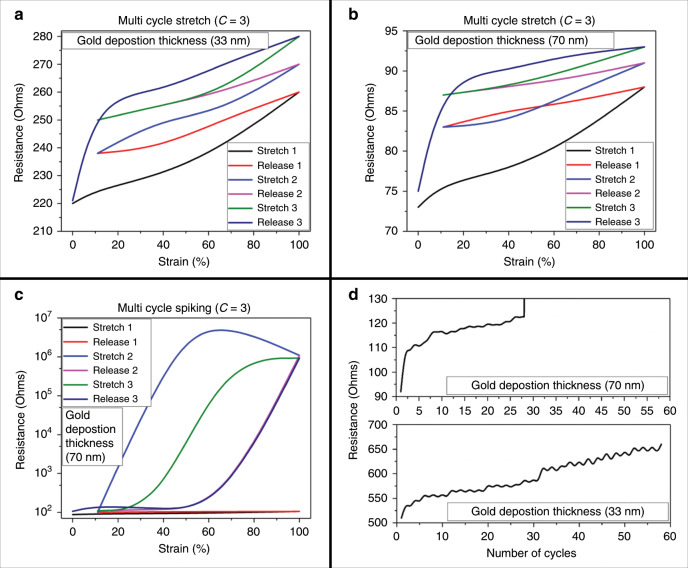
Multicycle hysteresis and reliability strain performance. **a**, **b** Multicycle hysteresis of the 33 nm (**a**) and 70 nm (**b**) Au coatings on the µserpentine. Looking only at these graphs, the performance of both coatings is similar. When examining the larger film fracturing samples from Fig. 4e, a large strain spiking signature can be seen (**c**). **d** Reliability measurements during fatigue testing for both the 33- and 70 nm Au coatings, further demonstrating the choice of 33 nm as an optimized coating thickness for this work.

In order to obtain fatigue limits of these coatings on the optimized µserpentines, repeated strains were applied to the 33 nm and 70 nm coated µserpentines. Figure 5a, b show the strain and release profiles for the 33 nm and 70 nm coatings (*C* = 3 cycles), respectively. These results are similar to a single-cycle results observed in Fig. 3. The three-cycle hysteresis curve shown in Fig. 5c illustrates irregularities observed in the 70 nm coated µserpentines. More than half of the tests performed at this thickness contain seemingly random spiking activity during any of its strain cycles. As mentioned previously in the EDS data of Fig. 4d and the associated SEM images of Fig. 4c, e, the 70 nm coating separates dramatically during strain, which directly contributes to the spiking seen in these cycles. Fatigue limit of both the 33- and the 70 nm coating on the optimized *α* = 10° µserpentine is depicted in Fig. 5d. The 70 nm coating is observed to fail under 30 strain cycles, whereas the 33 nm coating continues to perform up to 60 cycles of strain indicating approximately double the fatigue limit. These observed results were the rationale to pursue the 33 nm coating, which demonstrated a more reliable performance.

**Fig. 6:**
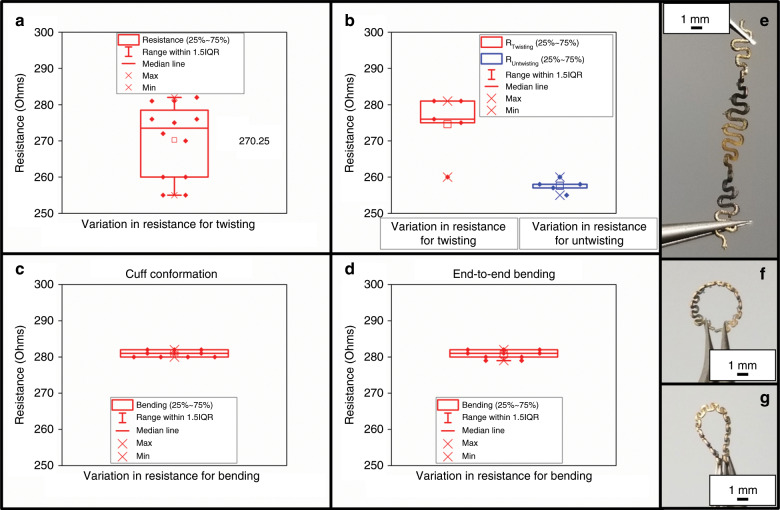
Conformational bending/twisting analysis of the optimized µserpentine with 33 nm Au coating. **a** Twisting conformation of the 33 nm Au-coated µserpentine, demonstrating a tight resistance signature for *N* = 11 twists. **b** A similar twisting signature plot, incorporating resistance values for *N* = 5 twisting and untwisting cycles, also demonstrating a tight grouping. **c** A cuff conformation resistance plot for the µserpentine, showing reliable performance after greater than *N* = 25 bends. **d** End-to-end bending of the µserpentine, also demonstrating a very similar tight grouping to **c**. **e** Optical image of twisting the µserpentine. **f** Optical image of the µserpentine in a cuff conformation. **g** Optical image of the end-to-end bending of the µserpentine.

Figure 6 demonstrates the electrical characterization through resistance measurements of the µserpentine base structure under twisting, bending, and conformation. Figure 6a shows the tight resistance distribution (mean of 275 Ω; +/−10.0 Ω) of the optimized µserpentine design as it is twisted for *N* = 11 cycles prior to becoming unusable. Figure 6b shows the slight variance of the resistance values (mean of 278 Ω; +/−8.62 Ω) during *N* = 5 twist cycles and the subsequent *N* = 5 turns to untwist the µserpentine (mean of 260 Ω; +/−1.82 Ω).

The variance from higher twisting to untwisting values is attributable to the relaxation of the gold coating and further demonstrates the reliability of the 33-nm coating. Figure 6c shows the resistance values (mean of 282 Ω; +/−0.88 Ω) for the cuff conformation of the µserpentine device, and Fig. 6d shows a very similar and tight grouping for the resistance values for an end-to-end bending conformation (mean of 282 Ω; +/−1.16 Ω). The images in Fig. 6e–g are micrographs of the twisting and bending conformations performed, with Fig. 6e representing data shown in Fig. 6a, b, Fig. 6f representing Fig. 6c, and Fig. 6g paired with Fig. 6d. The stability of the resistance in all of these conformations further lends credence to µserpentine optimization for a microelectrode application.

**Fig. 7:**
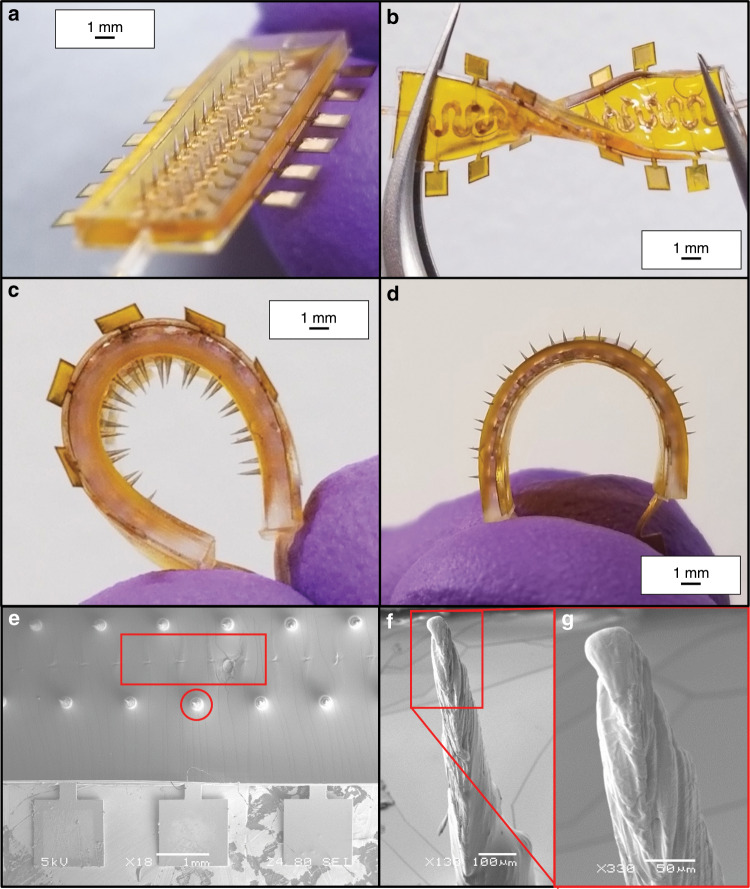
Optical and SEM images of packaged and assembled µserpentine sensor device. **a** Optical image of the fully assembled 3D microelectrode device, schematically represented in Fig. 1. **b** Optical image of the microelectrode µserpentine device undergoing twisting with a pair of tweezers. **c** Optical image of the 3D µserpentine microelectrode device undergoing end-to-end bending. **d** Optical image of the microelectrode µserpentine device in a reverse cuff conformation, exposing the microelectrode needle tips for imaging. **e** SEM image of the fully assembled device. The highlighted regions denote where the laser isolation trace is located beneath the PDMS layer (and hence are difficult to visualize) and where the exposed circular electrode tips emerge from the PDMS layer. **f** SEM image of the exposed electrode tip. After insulation, it is estimated that the electrode tips are 300 µm in height above the surface of the PDMS. **g** SEM close up of the electrode tip from **f**, highlighting the naturally formed µSLA striations, which contribute to the increased effective surface area of the 3D microelectrode.

Figure 7 demonstrates the fully integrated and assembled 3D printed, µserpentine 3D microelectrodes for biosensor applications. Figure 7a shows the design to device translation, representing a highly controllable and repeatable fabrication process. Figure 7b–d depict the flexibility of this fully encapsulated device (Kapton^®^ package and PDMS insulation) in several key conformations: twisting (Fig. 7b), cuff (Fig. 7c), and outward end-to-end bending (Fig. 7d). The device was robust enough to recover its original shape immediately, after the application of these flexural strains. The fabrication can be adapted potentially with a PDMS package and liquid EInGaN^35^ or cPDMS^36^ metallic traces to achieve a fully stretchable biosensor. As an initial step in device demonstration, Kapton^®^ was chosen due to its thermal stability, ease of defining through laser-micromachining, and good adhesion properties to deposited metal traces^37^. PDMS has been experimentally observed by our group, to be inhibited from curing at the interface between the resin and the elastomer. However, through the metallization of the 3D printed µserpentine (a necessary step in the device microfabrication), no issues with curing the PDMS layer on the 3D printed resin were observed. Figure 7e shows an SEM overview of the fully encapsulated and fabricated microelectrode device. The uninsulated electrode tips can be observed emerging from the PDMS, while the underlying µserpentine structure can also be observed owing to the conformal nature of PDMS coating and casting. The laser micromachined scribe line to isolate the electrodes from one another can similarly be observed. Figure 7f depicts a close-up SEM of the 3D printed, metallized electrode tips that are exposed above the PDMS insulation. Edge of this tip is shown in Fig. 7g, and the striations of the surface of the exposed tip can be viewed. The striations are formed in this way, due to the optimal µSLA printing conditions as detailed in Kundu et al.^38^, in which a 45° print angle was determined to be ideal for resolving the full electrode towers.

The versatility which 3D printing imparts on the fabrication process means that complex, arbitrarily defined 3D structures could be incorporated into the device, leading to a variety of potential lab-on-a-chip, wearable, and cell culture^39^ applications in areas, such as microneedles, 3D microfluidics, cellular constructs, and helices (Fig. S2). Using the fabrication process described in this work and a printer with smaller minimum feature sizes (such as Nanoscribe^40^ or Asiga Digital Light Projection printers^41^), smaller functional electrodes could be manufactured, potentially extending various surface geometries into the nanoscale. According to Widlund et al.^24^, the calculations used to optimize the printed µserpentines are ubiquitous due to the geometric nature of the key parameters of the device (*l*, *R*, *w*, *α*, etc.). However, practical design considerations, printing technique, and usability in packaged biosensor designs control the characteristics of µserpentines.

**Fig. 8:**
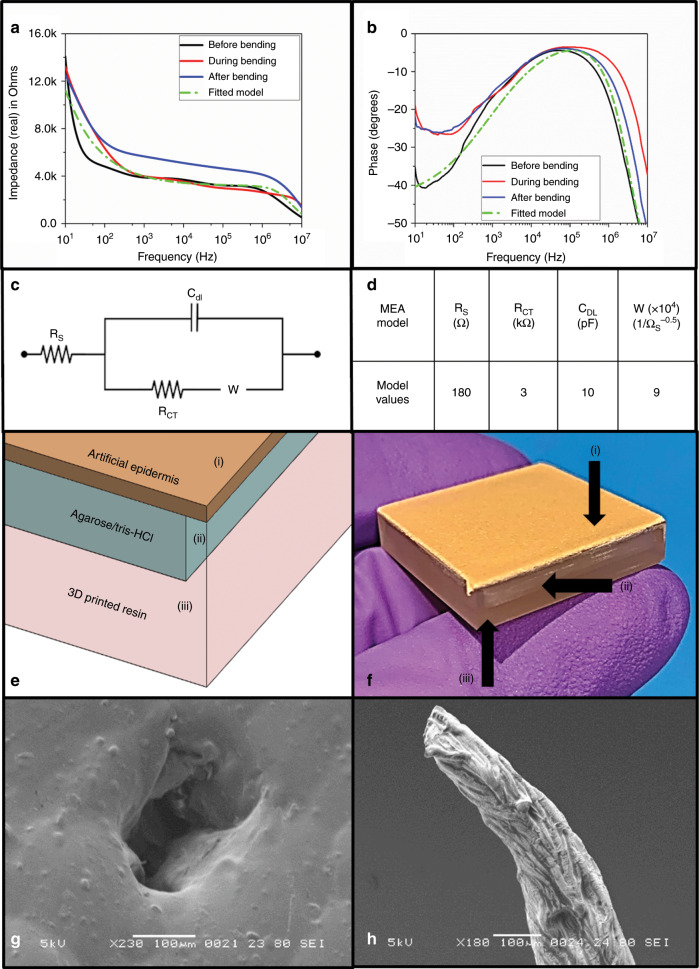
Characterization of the microelectrode µserpentine device and subsequent artificial epidermis/dermis transdermal electrical recording images. **a** Full spectrum impedance plot of the device before (black), during (red), and after (blue) bending, with a fitted impedance model (green) from which relevant circuit parameters were extracted. **b** Full spectrum phase graph for the device before (black), during (red), and after (blue) bending, with a fitted impedance model (green) from which relevant circuit parameters were extracted. **c** Representative circuit model for the microelectrode array profile extracted from **a**, **b**. **d** Extracted circuit parameters from the modeled circuit. **e** Schematic representation of the artificial skin model: (i) Corresponds to the artificial, non-conductive epidermis patch, (ii) the agarose/Tris-HCl artificial dermis tissue, and (iii) the 3D printed mold accommodating the skin model. **f** Optical image of the artificial skin model, with (i–iii) corresponding to the schematic components listed. **g** SEM image of a puncture site on the artificial epidermis, demonstrating the feasibility of the 3D printed microneedle microelectrode towers to penetrate skin for potential transdermal/tissue recording applications. **h** SEM image of an electrode tip from one device, which was not properly heat cured to give the resin its final robust structure. This is essential, or the resin electrodes will not be able to penetrate the skin or skin model.

Figure 8 compiles the electrical characterization of the 3D microelectrode µserpentine device. Figure 8a shows the full spectrum (10 Hz to 10 MHz) impedance of the electrode tips (~300 µm in height) before, during, and after bending strain was applied to the devices. At the electrophysiologically relevant frequency of 1 kHz^42^, the real part of the complex impedance was measured to be 4.2 kΩ (before), 4.6 kΩ (during), and 5.2 kΩ (after) (*N* = 3). These values demonstrate comparable 3D microelectrode characteristics to other reported approaches in literature, such as Guvanesen et al.^19^, where their average 1 kHz value is 7.6 kΩ (+/−2.20 kΩ). 3D printing high aspect ratio structures using µSLA processes exaggerates the striations that are visible in Fig. 7g. We anticipate the higher surface area created by these striations lowered the impedance values measured.

Typically to this end, nanomaterial electroplating or electroless plating has been one highly used method for increasing the surface area of microelectrodes, in order to help better extract biologically relevant data^43^. The micro-texturing inherent in 3D printing could aid in impedance reduction for these interfaces. Figure 8b represents phase values of full spectrum impedance before, during, and after the application of bending strain. The phase spectra depict characteristically microelectrode profiles^44^, starting as capacitive at phase between −20° and −40° and gradually becoming more resistive as the frequency increases, and the phase value approaches 0°. The phase data suggest a pattern observed by us^45^ and other microelectrode researchers^46^, in which the electrical profile of the mid-range frequencies is governed by a double-layer capacitance (C_DL_). The electrode–electrolyte interfacial impedance that occurs is dominated by the resistive elements of the phosphate buffer solution at higher frequencies, and as a result slightly more negative phase values are observed. An analytical microelectrode model is fitted to the real and phase parts of the impedance based on the circuit schematic shown in Fig. 8c, which represents the components of a complete MEA^44^: solution resistance (*R*_S_), charge transfer resistance (*R*_CT_), double-layer capacitance (*C*_DL_), and the Warburg element (*W*).

For extraction of the circuit parameters listed, Eq. 3 is solved:

3 $$Z(\omega ) = R_{\rm{S}} + \left[ {1/\left( {C_{{\rm{DL}}}(\omega )} \right. + \left\{ {1/\left( {R_{{\rm{CT}}} + W(\omega )} \right)} \right\}} \right]$$

The analytical model fits the experimental data well, demonstrating the impact of the components on the final electrical profile of the sputter coated, 3D µserpentine gold microelectrode. Figure 8d contains a table of the extracted values of the equivalent circuit for the analytical model that represents the combined three device states (before, during, and after bending).

In order to demonstrate a wearable 3D MEA sensor application, highlighting the versatility of the µserpentine design structure, an artificial skin model was developed in part with the protocol outlined in Besio et al.^47^. The agarose “dermal tissue” was cast in a custom 3D printed mold and covered with a non-conductive epidermal layer, as is represented schematically in Fig. 8e and optically in Fig. 8f. The surface direct current (DC) resistance of the agarose “dermal tissue” model layer was found to be ~60 kΩ (+/−1.40 kΩ; *N* = 3), and the end-to-end absolute resistance of the penetrated agarose was measured at 30 kΩ (+/−0.20 kΩ; *N* = 3). The µserpentine device electrodes were interfaced with the artificial skin model to record sub-dermal tissue DC resistance to confirm the ability of the microelectrodes to penetrate the epidermis (Fig. 8g). The device successfully acquired a reading of the underlying tissue model, at DC resistances between 40 and 50 kΩ, across several puncture points in the model. The resistivity of the model according to the resulting DC resistance measurements was calculated to be ~50 Ω-m, well within the expected values^47^. Generally, the 3D printed resin structures require heat curing after printing to ensure the full stability and rigidity of the fabricated structures^38^. As depicted in Fig. 8h, there was some damage noted to certain microelectrode tips after pressing on the artificial skin model. These tips may not have been completely thermally cured (usually the curing time for other device components serves well for this purpose), but reducing the tower aspect ratio could also help increase their stability after shorter heat curing times. This proof-of-concept demonstration highlights the potential for these sensors to be used in wearable electroencephalogram, electrocardiogram, electromyogram, and nerve conduction measurements^47^.

## Materials and methods

Thin Kapton^®^ (12.5 µm) sheets (DuPont™, USA) were laser micromachined to a size of 20 mm-by-5 mm, with 1 mm-by-1 mm extensions (6 on either side of the base substrate; 12 total) on which the microelectrode landing pads would be subsequently defined (Fig. 1a(ii)). Laser micromachined shadow masks were machined from 12.5 µm-thick 316L stainless steel (Trinity Brand Industries, USA) using the QuikLaze 50 ST2 laser micromachining system (Eolite Lasers, USA) with 1064 nm wavelength infrared (IR) laser light (6 mJ power, and 50 Hz repetition rate) (Fig. 1a(ii)). These masks served as stencils for subsequent metallization. Gold (5 N, 57 mm by 0.2 mm Au target; Ted Pella, INC., USA) was deposited on the 3D printed substrate (deposition voltage: 20 mV, and 13 nm/min deposition rate) to form 33 nm-thick packaging traces and landing pads through the Quorum Q150T Plus sputtering system (Quorum Technologies Ltd., UK) (Fig. 1a(ii)).

The 3D µserpentines with and without out-of-plane biosensor structures were designed with the Solidworks 2018 3D CAD software (Dassault Systems, USA) and 3D printed using commercially available clear (FLGPCL04) resins on the Formlabs Form 2 µSLA 3D printer (Formlabs, USA) (Fig. 1a, b(i)). The µserpentines were designed to be 400 µm-thick, and the pitch between the central points of the U-bends varies between the three printed designs, which are shown in Fig. 2c. The *α* = 0° design has a pitch of 1.5 mm; the *α* = −33° design has a pitch of 2.19 mm, and the *α* = 10° design has a pitch of 1.3 mm (Fig. 2c(i–iii)). All out-of-plane 3D printed structures were designed to be 400 µm at the base and with a height of 2 mm. The physical printed electrode cones before insulation resolved at approximately 1.1 mm above the µserpentine U-bend, and so the resolved electrode cones before insulation were 400 µm at the base and 1.1 mm in height. The pitch between the 3D structures was designed to be 1.3 mm (similar to the *α* = 10° design). The printed µserpentine was similarly metallized utilizing a sputter coater (Quorum Q150T Plus; Quorum Technologies Ltd., UK) with a layer of Gold (5 N, 57 mm by 0.2 mm Au target; Ted Pella, INC., USA), under the same deposition rates as outlined above (20 mV, and 13 nm/min deposition rate) to form conformal coatings across the entire 3D µserpentine structure with thicknesses ranging from 7 to 70 nm (Fig. 1a(iii)). The microelectrodes on the µserpentines were isolated down the center (both front and backsides) of the structure using the QuikLaze 50 ST2 laser micromachining system (Eolite Lasers, USA) with 1064 nm wavelength IR mode (6 mJ power, and 50 Hz repetition rate), which selectively ablated the gold and did not damage the resin (Fig. 1a(iii)). For the MEA design, the µserpentines were aligned with the terminated metal traces of the packaging substrate, and a small droplet of the Epo-tek^®^ EJ2189 silver-ink (Epo-Tech, USA) was placed on the µserpentines/trace interface to ensure connectivity. The package was allowed to cure for 24 h at 45 °C (Fig. 1a(iii)).

A drop-casted layer of 10:1 PDMS bulk polymer to curing agent (Slygard-184, Dow Corning, USA) was defined as the final insulation layer on the 3D electrodes (Fig. 1a(iii)). A custom designed and 3D printed mold was developed to assemble the devices into their final form factor. PDMS was cast within this mold to ensure a uniform thickness across the device. The assembly mold was sputter coated with gold separation layer to a thickness of 70 nm, to ensure curing of PDMS, which was observed by our group, to be inhibited by the resin. The thickness of the final PDMS insulation was defined and insulated at 1-mm thick (Fig. 1a(iv)). The device assembly was cured at 50 °C for an additional 24 h to attain the full crosslinked mechanical properties of the PDMS^48^.

Elongation experiments for µserpentine characterization were performed by clipping contacts to both ends of the µserpentines and recording the DC resistance measurements from a Keithley 2400 Sourcemeter (Tektronix, USA) (Figs. 3, 5, and 6). Twisting/bending analysis and hysteresis cycling were performed with tweezers under a stereoscope with wire leads epoxied to the landing pads for electrical characterization during the application of strain. Full-spectrum impedance measurements were performed with a BODE 100 Impedance Analyzer (Omicron Labs, Austria) with a Platinum (Pt) anode in Dulbecco’s Phosphate Buffered Saline (1×) (Gibco, USA; Fig. 8a, b). For SEM imaging of samples, the Zeiss Ultra-55 SEM (Zeiss, Germany) was used, and EDS was performed on the same SEM with the Noran System 7 EDS with Silicon Drift Detector X-ray Detector (Thermo Fisher Scientific, USA) (Figs. 2, 4, 7, and 8). All optical images were obtained with an iPhone XS (Apple, USA). Data graphing was performed in Origin 2016 (OriginLab Corporation, USA). Data fitting and impedance modeling were performed in MATLAB R 2018b (Mathworks, USA). Effective stiffness and normalized maximum tensile strain calculations were defined and implemented with appropriate design values using Wolfram Mathematica 11.3 (Wolfram, USA) (Fig. 2a, b). The Agarose dermal tissue model was created from a 1 M solution of Tris-HCl (pH 6.1) and powdered agarose as per Besio et al.^47^. The mixture was placed in a beaker and stirred continuously to a boil of 100 °C on a hotplate. According to the refs. ^47,49,50^, 3 mm-thick agarose mixed in the aforementioned protocol models dermal tissue closely, with a conductivity of 0.06 S/m. The agar was poured into a custom 3D printed 25 mm-by-25 mm mold (3 mm-thick as per the protocol from these papers) and allowed to fully crosslink at 25 °C for an hour. The epidermis was modeled with an artificial, 500 µm-thick epidermal patch (Remedy Simulation Group, USA) and attached to the mold/agarose dermis with integrated adhesive. The artificial epidermal patch is non-conducting to model an enhanced effect of the dead skin layer of the stratum corneum present in real epidermal skin layers. 3D MEAs (depicted in Fig. 7a) were pressed onto the artificial epidermis/dermis skin model and DC resistance values were obtained from the 3D electrodes across the device using a Keithley 2400 Sourcemeter (Tektronix, USA) (Fig. 8f).

## Conclusions

We have explored the capabilities and limitations of 3D µSLA printed µserpentine for their applicability to 3D microelectrodes. We have further integrated such metallized µserpentines with a Kapton® package and a PDMS insulated to develop a dynamic 3D MEA. The µserpentine base structures used were optimized according to two key compound equations for the effective stiffness and maximum U-bend strain. The final optimized µserpentines had an *l*/*R* ratio of 2, and an *α* of 10°, creating a µserpentines that could stretch up to 155% its resting length. This optimized µserpentine was subsequently characterized with varying conformal gold coating thicknesses, to find the optimal thickness to retain resistance values during strain. The resulting coating thickness was found to be 33 nm and performed equally as well over twisting and bending strain analyses and with good reliability over 60 strain cycles. The final optimized and coated µserpentine structure was integrated into a device package built on PI (Kapton®) substrates with metallized traces to connect to the laser isolated 3D microelectrode and encapsulated with PDMS insulation. The 3D microelectrode device was characterized for impedance and phase over a full frequency spectrum (10 Hz to 10 MHz), and the resulting electrophysiologically relevant 1 kHz values were measured for a dynamic MEA application: 4.2 kΩ (before bending), 4.6 kΩ (during bending), and 5.2 kΩ (after bending). This device was then employed to procure transdermal readings across an artificial agarose skin model, measuring the expected resistivity of 50 Ω-m. This details the capabilities, limitations, and the versatility of µSLA printed serpentine-based 3D microstructures for various sensor devices with applications in wearable technologies, as well as dynamic cell culturing and in vitro conditions.

## Supplementary information

Supplemental Article Material

Editoiral Summary
