# Supplementary material for: Capabilities and limitations of 3D printed microserpentines and integrated 3D electrodes for stretchable and conformable biosensor applications
Source: Microsyst Nanoeng. 2020 Apr 20;6:15. doi: 10.1038/s41378-019-0129-3 (PMC8433388; doi:10.1038/s41378-019-0129-3)
Supplement: Supplementary file 1 — Supplemental Article Material [file 41378_2019_129_MOESM1_ESM.docx]

**Capabilities and Limitations of 3D Printed Microserpentines, and Integrated 3D Electrodes for Stretchable and Conformable Biosensor Applications**

**Charles Didier^1,2^, Avra Kundu^1^, and Swaminathan Rajaraman^*1,2,3,4^**

*^1^Nanoscience Technology Center (NSTC), ^2^Burnett School of Biomedical Sciences,^3^Department of Materials Science & Engineering,^4^Department of Electrical & Computer Engineering; University of Central Florida, Orlando, FL 32828, USA*

A sample optogenetic stimulator / µLED indicator device was later created by using ink-casted traces bridging µLEDs between two optimized serpentines. The µLED stimulator device was fabricated in the same fashion as described in the materials and methods section, but using two µSLA 3D printed µserpentines on a Kapton^®^ package. A stencil mask for conductive ink traces for the µLED stimulators was micromilled (T Tech, QC J5, USA) from 50µm 316L stainless steel (Trinity Brand Industries, USA), and the traces for the µLED indicator device were defined by casting Epo-tek^®^ EJ2189 silver-ink (Epo-Tech., USA). Conductive ink was cast on the µserpentines to ensure uniform connectivity between both µserpentines. Kingbright µLEDs (1mm x 650µm x 350µm) (Mouser Electronics, USA) were used in the µLED stimulator demonstration (figure S1(b & c)), and the total device was allowed to cure for 48 hours at 45ºC, to create a strong adherence to the Kapton package and ink cast µserpentines (figure S1a).

Figure 1c outlines the various geometric aspects of a µserpentine which were incorporated into the analytical model for optimization of the final serpentine design, while figure S1a demonstrates the process flow schematic for the application of this design to a fully packaged µLED device. Figure S1(b & c), demonstrate the fully fabricated and functional optogenetic stimulator / µLED device, being strained in different conformations. The device was fabricated as depicted in figure S1a, and still remained robust even after twisting and bending strains exceeding N=25 cycles.

Other non-traditional shapes can be monolithically integrated to the base serpentine design such as µhelices, microfluidic ports etc., as should be evident from the versatile proof of concept, fabricated constructs demonstrated in this work (figure S2). The non-traditional out of plane structures demonstrated here were created after the full characterization of the µSLA µserpentines, on both a µSLA and Digital Light Processing (DLP) 3D printer. Figure S2(a-c), shows SEM images of the sample cell surface adhesion promoter, the µhelices, and a sample integrated microfluidic port. Figure S2(d-f), illustrates the same sample constructs on the µserpentine, however the clear difference in the resolution of these structures demonstrate the use of Digital Light Processing (DLP) 3D printing (Asiga Ltd., Australia) to fabricate these devices.

**
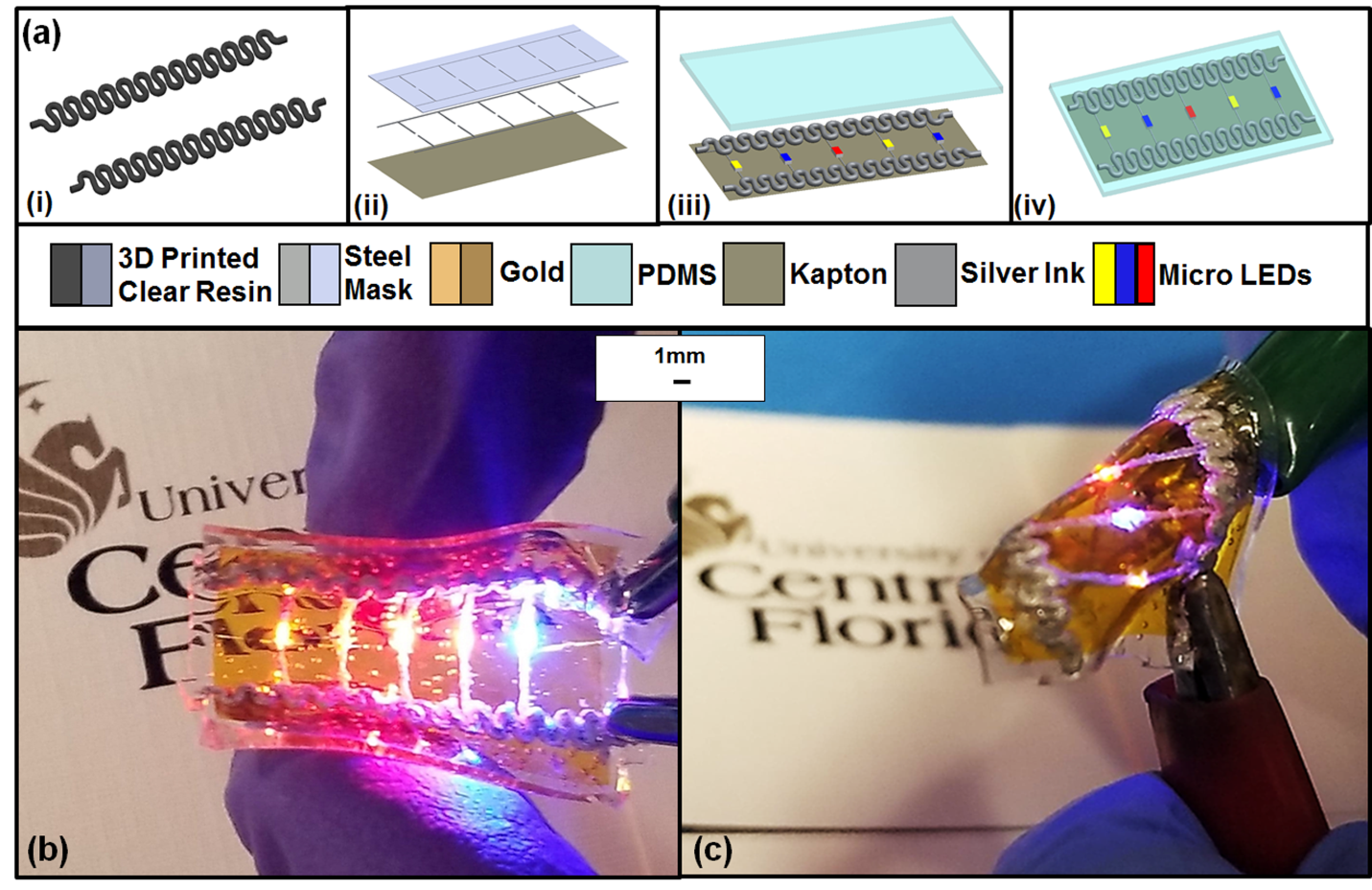
**

Figure S1: Additional device demonstration, created with the optimized serpentine parameters: (a) Schematic representation of a µserpentine µLED device. (i) Schematic of a 3D printed double µserpentine conformation, where one µserpentine would correspond to the anode and one to the cathode of the device. (ii) Schematic of the ink-casting process on a laser micromachined Kapton® substrate. (iii) Assembly of the schematic device, illustrating the positioning of the two ink-coated µserpentines onto the substrate, the µLED addition, and the PDMS encapsulation. (iv) Schematic of the final fully assembled device. (b) Optical image of the assembled and fabricated device, with µLEDs lighted under a concave bending conformation. (c) Optical image of the µLED device continuing to perform successfully even while twisted into a tight barrel conformation.

**

**

Figure S2: SEM images of alternative out of plane, monolithically integrated structures on µserpentines: The intention for these images is to illustrate the potential for several microsensors integrated out of plane from µserpentines: (a-c) SEM images of µSLA 3D printed cell surface adhesion promoters (a), µhelices (b), and microfluidic ports (c). (d-f) SEM images of the same 3D printed cell surface adhesion promoters (d), µhelices (e), and microfluidic ports (f), however these were printed on a DLP 3D printer.
