# Supplementary material for: Capabilities and limitations of 3D printed microserpentines and integrated 3D electrodes for stretchable and conformable biosensor applications
Source: Microsyst Nanoeng. 2020 Apr 20;6:15. doi: 10.1038/s41378-019-0129-3 (PMC8433388; doi:10.1038/s41378-019-0129-3)
Supplement: Supplementary file 2 — Editoiral Summary [file 41378_2019_129_MOESM2_ESM.zip]

# *Microsystems & Nanoengineering*

Wearables: Stretchable Microstructures for Microelectrode Sensors

A unique method to make wearable electronics using flexible microstructures have been explored and characterized by researchers in the United States. Wearable devices such as sensors or power generators require stable, stretchable electronics, otherwise their electrical performance will degrade when they are deformed. Swaminathan Rajaraman’s team at the University of Central Florida investigated the capabilities and performance of 3D-printed microserpentines; shapes engineered to be flexible. They used an analytical model to optimize the design of the microserpentine structures to be maximally flexible. Next, they applied varying thicknesses of nano-gold to the microserpentines to determine which thickness remained most stable when stretched. Finally, the team incorporated the microserpentines into a sensor device to take measurements from artificial skin. These findings demonstrate the potential of 3D-printed microserpentine structures for use in sensors and other wearable electronics.

Total word count: 134

1^st^ sentence characters with spaces: 149

Related article manuscript number: MICRONANO-00923R

Article title: Capabilities and Limitations of 3D Printed Microserpentines, and Integrated 3D Electrodes for Stretchable and Conformable Biosensor Applications

Corresponding author and affiliation/s: Swaminathan Rajaraman, University of Central Florida, United States

**About your Editorial Summary — please read**

**Before approving this Editorial Summary, please carefully check that (1) the summary text lists the correct author(s) and (2) the spelling and order of all author names and affiliations are correct.**

This **Editorial Summary** is based on your manuscript that was recently accepted for publication in *Microsystems & Nanoengineering*. It provides a non-specialist audience with a synopsis of your key research outcomes and conclusions. This value-added service provided by Springer Nature is designed to raise interest in your research across the broader community.

Springer Nature will publish the summary on the journal’s website, and it will be freely available under a under the CC BY licence (Creative Commons Attribution v4.0 International Licence) (see the journal website for details). We encourage you to re-use the summary to bring attention to your research; for example, you can host it on your own website and share it via social-networking platforms. Please attribute the summary to *Microsystems & Nanoengineering* and your article (e.g. by providing a link to your article) and do not make derivatives.

Please note that to maximise the usefulness of these summaries they must follow several stringent guidelines:
-- Spelling, punctuation and style are set according to *Nature* editorial guidelines. As this summary is aimed at non-expert readers, some concepts and technical terms will be simplified.
-- Total length must be no more than 135 words. It is likely that not all points in the paper will be covered.
-- The first sentence must be no more than 280 characters, including spaces, to allow use on microblogging sites.
-- The headline must consist of a brief generic subject identifier followed by a short description. No more than 10 words in total.

Please contact the editorial office ([mems_nano@mail.ie.ac.cn](mailto:mems_nano@mail.ie.ac.cn)) immediately with corrections should you find any factual errors in this Editorial Summary.
